# Supplementary material for: Are carbon emissions trading and green financial instruments synergistic? -Comprehensive quantitative research based on content analysis
Source: PLoS One. 2024 Mar 7;19(3):e0298601. doi: 10.1371/journal.pone.0298601 (PMC10919601; doi:10.1371/journal.pone.0298601)
Supplement: S1 Data — (ZIP) [file pone.0298601.s001.zip › Data/地级市Z_F工作报告.zip!/╦─┤¿/╤┼░▓╩╨/└┤╫╘╣1⁄2╓┌║┼í╛┬φ┐╦╩2╛▌═°í┐_2015.docx]

各位代表：

　　现在，我代表市人民政府，向大会作政府工作报告，请予审查，并请市政协各位委员提出意见。

2014年工作回顾

　　2014年是雅安发展史上克难奋进、成果丰硕的一年。在市委的坚强领导下，在市人大、市政协的监督支持下，市人民政府认真落实市委三届七次全会的决策部署和市三届人大四次会议的决议，全面完成了全年目标任务。全市灾后恢复重建有力有序有效推进，经济呈现持续回升、稳步向好态势，社会事业全面进步，人民生活得到改善，社会大局保持稳定。纳入灾后恢复重建总体规划的2160个项目，累计完工1096个，完工率50.7%;累计完成投资411亿元，占估算总投资的57.6%，成功实现项目完工率和投资完成率“双过半”目标。实现地区生产总值462.41亿元，增长11%，增速居全省第1位;一般公共预算收入27.38亿元，增长19.4%;农民人均纯收入9056元，增长11.9%;城镇居民人均可支配收入24435元，增长9.8%。

　　一年来，我们紧紧扭住重建中心，牢牢把握民生优先、科学重建主线，严格执行重建总体规划和专项规划，统筹把握重建重点和关键环节，灾后恢复重建有力有序有效推进。

　　城乡居民住房和公共服务设施重建优先推进。坚持把城乡居民住房重建作为首要任务加快实施，全市农房重建全面完成，城镇居民住房重建完成33.7%。其中：625户特困群众由政府“兜底”建保障房，于2014年春节前实现搬新家过新年;对4482户困难群众建房实行“一对一”帮扶，于2014年4月20日前实现搬迁入住。坚持优先推进公共服务设施重建，累计完工项目642个、完工率61.8%。其中：学校完工239所、完工率76.6%，医疗卫生项目完工73个、完工率79.3%，分别超过重建平均进度25.9、28.6个百分点。就业和社会保障、科技、体育、社会福利、残疾人康复托养等重建项目进展顺利。雅安中学高中校区、雅安职业教育发展区、市医疗服务中心、市文化中心等重大项目加快建设。

　　城乡基础设施和城镇体系重建有序推进。坚持把恢复功能与提升水平有机结合，积极推进基础设施重建。累计完工项目44个、完工率46.8%。雅康高速公路和国道351线、318线、108线重建项目快速推进，5条重要经济干线建设进展顺利。水利、能源、通信等重建项目加快实施。按照“中心带动、轴线聚集、县城提升、整体推进”的思路，加快推进城镇体系重建。主城区、6个县城以及16个重点镇重建项目有序实施，城镇环卫、应急避难场所等重建项目顺利推进，供排水、电力、广电、消防和污水垃圾无害化、资源化处理设施逐步完善。

　　产业重建抓住机遇加快推进。着眼雅安长远发展和群众致富奔康，用好用活用足各类支持政策，统筹推进产业重建。芦天宝飞地产业园区(雅安经开区)、芦山产业集中区、荥经循环经济示范园区等工业园区基础设施和配套功能不断完善;总投资30亿元的恒天汽车项目和总投资12.6亿元的建安车桥扩能项目开工建设，王老吉、雅化、三九、金红叶等项目加快推进。雅安国家农业科技园区、芦山现代生态农业示范园等农业园区加快建设，百里茶产业、百里猕猴桃产业和百里花果金果产业带建设初具规模。国家生态文化旅游融合发展试验区项目有序推进，旅游景区景点重建项目加快实施。

　　地质灾害防治工作扎实有效。把地质灾害防治作为“生命工程”贯穿灾后恢复重建始终，全面完成冷木沟、教场沟等235个重大地质灾害治理工程、387处地质灾害排危除险工程、107处受损修复工程并纳入日常管护。健全“三本台账”，强化动态防治和监测预警，全面完成3330户地灾避让搬迁安置，成功实现安全度汛和地质灾害“零伤亡”。自然生态系统修复、自然保护区和森林公园、生态基础设施等重建项目进展顺利，大熊猫等珍稀濒危物种保护得到加强。

　　工程质量和资金安全得到有力保证。坚持进度服从质量，严格落实“三避让”原则和抗震设防要求，强化技术指导和建材质量监管，拉网式检查重建项目128次，整改问题625个，重建工程质量得到有效保证。明确重建项目分级管理责任，优化项目审批流程，强化对招投标活动的监管，加大对违法违规行为的处罚力度;制定灾后恢复重建资金使用管理办法，强化全程监督、跟踪审计，确保了阳光重建、廉洁重建。

　　积极拓展援建合作，7个援建市对口帮扶的22个援建项目已完工6个，到位援建资金3.2亿元;7个省级部门帮助我市招商引资签约项目20个、总投资196亿元。

　　过去的一年，面对繁重的重建任务和复杂的各类矛盾，我们认真践行以地方为主的灾后恢复重建机制，严格落实“五总”要求，坚持目标倒逼、责任倒逼、结果倒逼，在“赶考”中前行，在“硬仗”中攻坚，促进了重建整体提质提速，成功实现了“4·20”重建项目全面开工、“7·20”重建项目全部开工、年底项目完工率和投资完成率“双过半”以及农房重建全面完成的既定目标。始终把灾后恢复重建与经济社会发展有机结合、统筹推进，以项目建设稳定投资增势，以产业重建增强“造血功能”，着力放大重建效应，灾后恢复重建对全市经济社会发展的促进作用正在逐步显现。习近平总书记、李克强总理等党和国家领导人分别作出重要批示，充分肯定芦山地震灾后恢复重建工作，这是对我们莫大的鼓舞和鞭策。

　　我们将再接再厉，朝着既定的目标，在全面建设灾后幸福美丽新家园的征程上奋勇前行!

　　一年来，我们主动适应经济发展新常态，积极应对宏观经济下行压力，着力转化比较优势，全力实施后发追赶，全市经济呈现持续回升、稳步向好态势。

　　经济保持稳定增长。及时出台稳定经济增长12条措施，以及促进工业、建筑业发展等配套措施，着力支持实体经济发展，下大力气解决经济运行中的突出问题和深层次矛盾，保持了经济持续较快增长。抓好投资稳增长，全社会固定资产投资完成471.4亿元，增长33.6%，增速居全省第3位。雅安人民期盼已久的成雅铁路(川藏铁路成都朝阳湖至雅安段)启动建设，飞仙关电站投产发电，大岗山电站一期下闸蓄水，永定桥水利枢纽工程基本建成并下闸蓄水，铜头引水、九龙水库、南郊水厂引水工程加快实施。抓好工业稳增长，综合运用电力、金融等扶持政策，稳定工业经济增势，规模以上工业增加值增长12.5%。抓好农业稳增长，在特色化、产业化和品牌化上下功夫，第一产业增加值增长4.6%。抓好服务业稳增长，建立新兴先导型服务业发展推进机制，促进服务业健康发展，服务业增加值增长10.6%;旅游综合收入突破百亿大关，达到108.6亿元，增长53.6%。抓好建筑业稳增长，支持建筑企业做大做强，建筑业增加值增长22.7%。抓好消费稳增长，积极搭建各类消费平台，消费市场得到有效拓展，全社会消费品零售总额增长13.3%。

　　产业发展步伐加快。突出“工业挑大梁”，启动实施“千亿产业培育计划”，水电及矿电结合产业产值达到232.1亿元，机械装备制造业产值达到61.4亿元，农产品加工业产值达到62.5亿元，新材料新能源产业产值达到60.4亿元。新增规模以上企业20户、国家高新技术企业和创新型企业14户，主营业务收入上亿元企业达到94户。夯实“农业强底座”，现代农业加快发展。稳定粮食播面，粮食产量46.96万吨。重点培育“5+3”优势特色产业，新发展茶叶、果蔬、中药材等产业基地30.5万亩。强化农产品质量品牌建设，完成国家茶叶产品质量监督检验中心建设，新增有机生产基地3万亩、绿色生产基地10.3万亩，认证有机食品37个、绿色食品12个;新增国家地理标志产品1个、四川省著名商标7件。成功组建蒙顶山茶叶股份有限公司，新增市级以上产业化龙头企业10户、农民专业合作社218个。转移农村劳动力35.65万人，实现劳务收入43.78亿元。狠抓“三产当尖兵”，以生态文化旅游业为龙头的现代服务业加快发展，碧峰峡和蒙顶山景区创国家5a级旅游景区工作全面启动，熊猫古城和金凤山景区升级为国家4a级旅游景区。成功举办第七届中国·雅安动物与自然国际电影周、第十届蒙顶山国际茶文化旅游节等活动。雅安市物流园区、川滇藏商贸物流园区等现代物流项目加快建设，康养、金融、房地产、保险、运输、通讯等服务业加快发展。

　　县域经济提速发展。把发展壮大县域经济摆在更加重要的位置，研究制定加快县域经济整体跨越的意见，促进“三大片区”整体联动、特色发展，全市6个县全部纳入全省扩权强县改革试点。注重优势转化，加快产业发展，做大县域经济规模，6个县的地区生产总值、一般公共预算收入、全社会固定资产投资分别增长12.3%、28.1%和28.6%。落实鼓励民营经济发展各项政策措施，激发民营企业的活力和创造力，全市民营经济增加值占地区生产总值比重达58.3%。

　　一年来，我们始终坚持民生优先，着力办好民生实事，全面发展社会事业，社会大局保持和谐稳定。

　　民生实事全面完成。年初确定的“九项民生工程”、“20件民生实事”全面完成。一般公共预算支出中民生保障支出占65.8%。

　　社会保障不断完善。突出抓好就业促进和社会保障工作，城镇新增就业19690人，城镇登记失业率3.84%;城市居民最低生活保障标准提高到每人每月330元，农村居民最低生活保障标准提高到每人每年1980元;城乡居民养老保险覆盖43.65万人;“五保”对象集中供养率达到61%;新农合参合率达到99.6%;城镇居民医保参保率达到97%，实现与全省并网医院互联互通;城乡医疗救助144378人次，城乡居民健康档案规范化电子建档率居全省第一。开工建设保障性住房和实施危旧房棚户区改造8902套、竣工4478套。强化扶贫攻坚，精准扶持14950名农村扶贫对象改善生产生活条件。妇女、儿童、老龄、残疾人、慈善、红十字等工作扎实推进。

　　社会事业全面发展。义务教育普惠政策得到全面落实。“三免一补”工程扶助学生27.4万人次，营养改善计划惠及9.89万农村义务教育学生，资助普通高中和中职教育家庭经济困难学生2.5万人。重视农村学校特别是中高山区、民族地区学校建设，补充农村教师455名。全市普通高中高考一本上线人数增长22%。健全市、县、乡、村四级医疗卫生服务体系，基本公共卫生均等化取得新进展，国家基本药物制度在公办医疗卫生机构全面实施。市广电中心基本建成，陆家坝体育活动中心完成主体工程建设。组团参加全省第十二届运动会和首届农民艺术节、第七届少数民族艺术节并取得优异成绩，仁川亚运会雅安籍运动员勇夺2金1银。群众性文化体育活动蓬勃开展。文化遗产得到有效保护。启动实施“单独两孩”政策，人口自然增长率2.82‰。第三次全国经济普查顺利完成。国家安全、国防动员和民兵预备役工作进一步加强，省级双拥模范城市创建通过迎检考评。民族、宗教、防震减灾、档案、保密、科协、地方志、社会科学等工作扎实推进，外事、侨务、对台工作取得新进展。

　　社会治理得到加强。深入推进依法治市，“六五”普法扎实开展。依法全面履行政府职能，加强安全生产和市场监管，着力开展矿山、交通、消防、食品药品等重点行业和领域专项整治，遏制了重特大事故发生。深化反分裂、反渗透、反恐怖斗争和地震灾区、水电库区、涉藏地区稳定工作，社会大局保持稳定。强化平安雅安建设，依法严厉打击各类违法犯罪，社会治安秩序良好，群众安全感、满意度持续提升。基层政权建设不断强化，村(居)活动阵地建设得到加强，村(居)民自治水平进一步提高。扎实推进“法律进重建、调解进项目”活动，集中解决了一批关系群众利益、影响社会稳定的矛盾纠纷。

　　一年来，我们着力统筹城乡建设发展，全市新型城镇化步伐不断加快，新农村建设扎实推进，生态建设持续加强。

　　城乡规划不断完善。《雅安市城市总体规划(2013-2030)》编制完成并获省政府批准，雅安城市性质定位为川西枢纽和区域性中心城市、国际化旅游生态城市、四川省级历史文化名城。《大兴片区控制性详细规划》等7项城市规划、《2014-2020雅安市中心城区通信基础设施专项规划》等3个专项规划，以及81个乡镇总体规划编制顺利完成，重点景区和特色村规划编制正式启动。

　　城乡面貌逐步改观。投入资金36.44亿元加快城市建设。旧城改造有序推进，主城区“一城两线三出口”提升工程全面展开，姚桥新区城市功能不断提升，大兴片区组团启动建设。雨名通道建设全面启动，雨城区、名山区、芦天宝飞地产业园区(雅安经开区)产城一体化发展取得新进展。北外环线(主城区绕城路)加快建设，沿江公路改造基本完成，青衣江内环线基本贯通。青年路、和平路、金凤景观大道北段、周公河左岸等9条市政道路建设全面完成，大兴大桥形成通车能力。姚桥滨河公园、北纬30度公园等建成投入使用。县城建设力度加大，全省“百镇建设试点行动”加快实施，首批35个幸福美丽新村试点扎实推进，灾后重建新村聚居点基本建成，3个新农村综合体全面建成。雨城、汉源、石棉3个省级新农村示范县(区)加快建设。

　　人居环境不断改善。深入开展城乡环境综合治理，主城区拆除违章建筑8万平方米。强化重点区域市容环境综合整治，城市“五乱”现象得到有效遏制，主城区生活垃圾无害化处理率达到98%;修复城市病害路段10公里，强化易堵路段交通秩序管理，人力客三轮整治取得阶段性成效，城区通行能力得到改善;关闭截流青衣江沿岸直排点20个，新建污水管网20.8公里，主城区污水处理率达到88.6%。省级文明城市和国家级卫生城市创建工作有序开展，智慧城市建设加快推进，成功申报全国ic卡多应用推广示范城市。

　　生态建设持续加强。有序推进全国生态文明示范工程试点工作和天然林保护工程国家级示范市建设，雅安成功纳入国家首批生态文明先行示范区。持续实施“三大生态工程”，强化长江上游生态屏障建设，森林覆盖率达到62.86%。大相岭自然保护区升级为省级保护区，栗子坪自然保护区被授予大熊猫野化培训放归基地。全市大气环境质量持续改善，空气质量居成都经济圈城市群首位。医疗废弃物实现集中处置。农村面源污染得到有效整治。严格环境保护“三同时”制度，强化产业园区企业准入门槛和生产过程监测，淘汰17户企业落后产能。万元地区生产总值能耗和万元工业增加值能耗下降完成省定目标。

　　一年来，我们深入推进重点领域和关键环节改革，着力提高开放合作实效，有力地激发了发展的内生动力和市场活力。

　　重点领域改革深入推进。坚持简政放权，加快行政审批制度改革，取消、调整、下放行政审批事项96项。深化国企改革，雅安茶交所股权多元化改革加快实施。深化金融领域改革，成功组建雅安农村商业银行，有序推进雅安市商业银行增资扩股工作，引进浦发银行在雅设立分行，雅安雨城惠民村镇银行挂牌成立。深化财税体制改革，深入推进增值税改革，完善财政转移支付办法，调整全市分税制体制以及企业所得税、契税、印花税分享体制。深化农村改革，农村土地承包经营权确权试点工作顺利推进，农村房屋所有权确权登记颁证和城乡建设用地增减挂钩试点工作启动实施，农村集体建设用地使用权确权工作基本完成。深化资源要素配置市场化改革，水、电、气等领域改革稳妥推进。深化行政管理体制改革，进一步理顺了市、区管理体制，重点完善了芦天宝飞地产业园区(雅安经开区)管理体制。改革工商登记制度。推进政府机构改革，卫生计生、文化体育、商务粮食、食品药品等机构完成职能整合，工商管理、质量技术监督、城市管理等体制改革基本完成，事业单位改革加快推进。

　　开放合作步伐不断加快。抢抓“1+7”同城化发展机遇，拟订成都经济区区域合作项目清单，与眉山市签订《区域合作框架协议》，与乐山市等6个市(州)签订《大峨眉区旅游产业跨越发展战略合作协议》，与双流县签订《区域合作备忘录》，与天府新区成都直管区开展金融等领域战略合作。强化与攀枝花市、凉山州的合作，加快攀西战略资源创新开发试验区建设。制定加强投资促进工作的意见，组团参加第十五届西博会、“中外知名企业四川行”、中国(攀枝花)钒钛产业博览会、全省首届旅博会等重大投资促进活动，招商引资取得实效。全年引进到位市外资金290亿元，增长13.9%。引进投资5亿元以上项目16个、投资10亿元以上项目5个;实际利用外资2066万美元。

　　一年来，我们以党的群众路线教育实践活动为载体，着力加强政府自身建设，政府的执行力、公信力和依法行政能力得到有效提升。按照中央、省委和市委的统一部署，深入开展政府系统党的群众路线教育实践活动，着力解决了“四风”方面存在的突出问题。自觉接受市人大及其常委会的法律监督、工作监督和市政协的民主监督，认真办理人大代表建议、批评、意见130件和政协委员提案183件，办结率100%。认真听取民主党派、工商联、无党派人士和人民团体的意见，虚心接受群众监督和社会舆论监督。严格遵守中央“八项规定”、省委省政府“十项规定”，将政府机关公务用车、办公用房、公务接待等逐一规范到位，市级“三公经费”同比下降22.84%。扎实开展政府系统“止懒治庸”专项整治活动，行政效率进一步提高。强化对重点领域、关键岗位的行政监察和审计监督，严肃查处了一批违法违纪案件。

　　各位代表，刚刚过去的2014年，我们在极其艰难、极具挑战的严峻形势下，团结拼搏、感恩奋进，付出了辛劳和汗水，收获了成功与喜悦，迈出了后发追赶的坚实步伐!成绩来之不易，这是党中央、国务院，省委、省政府坚强领导、亲切关怀的结果，是市委统揽全局、科学决策的结果，是市人大、市政协履职监督、共同努力的结果，是省级部门、兄弟市(州)和社会各界关心帮助、倾力支持的结果，是全市干部群众齐心协力、共同奋斗的结果。在此，我谨代表市人民政府，向全市人民，向所有关心、支持和参与雅安建设发展的各界人士，表示衷心的感谢，并致以崇高的敬意!

　　在看到成绩的同时，我们也清醒地认识到：灾后恢复重建工作中还存在进度不平衡、管理不到位等问题，重建水平有待进一步提高。随着重建进入中后期，各项要求会更高更严，多种原因积淀的一些矛盾正逐步显现，安全生产、地灾防治、用地保障等仍将面临巨大的考验。雅安经济社会发展欠账多，产业化程度不高，比较优势和发展潜力没有得到有效转化和释放，新型工业化和新型城镇化短板明显，实现“同步奔康”任务艰巨。灾区、库区、涉藏地区交织叠加带来的影响社会稳定的因素较多。教育、医疗、养老等公共服务与群众期待还有较大差距，扶贫攻坚任务仍然艰巨。部分干部思想观念不够解放，行政效能还需提高，政府自身建设有待进一步加强。这些问题，我们将高度重视，并努力在今后的工作中切实加以解决。

2015年目标任务和主要工作安排

　　今年是灾后恢复重建的攻坚之年，也是全面完成“十二五”规划的收官之年。当前，经济发展步入新常态，压力与动力并存，机遇和挑战同在。我们既要深刻认识新常态带来的发展新趋势、新规律和新要求，主动适应速度变化、结构优化和动力转换的新特点，也要科学分析市情、吃透市情，进一步找准定位、把握机遇、转化优势、激发活力，努力实现后发追赶。只要我们坚定发展信心，保持发展定力，完全能够在新常态下实现新的更大作为。

　　今年政府工作的总体要求是：认真贯彻落实党中央、国务院，省委、省政府和市委的决策部署，按照本次会议确定的目标任务，主动适应经济发展新常态，坚持重建攻坚，坚持后发追赶，放大重建效应，专注潜力转化，为三年全面完成灾后恢复重建任务打下决定性基础，保持经济平稳健康发展和社会大局稳定。

　　今年全市灾后恢复重建主要工作目标建议：到7月20日，规划重建项目完工率、投资完成率均达80%;到12月31日，规划重建项目完工率、投资完成率均达90%。

　　今年全市经济社会发展主要预期目标建议：地区生产总值增长9%;全社会固定资产投资增长12%;社会消费品零售总额增长12%;一般公共预算收入增长8%;城镇居民人均可支配收入增长9.5%，农民人均纯收入增长11%;城镇登记失业率控制在4%以内;人口自然增长率控制在5‰以内;城镇化率提高1.2个百分点;完成省上下达的节能减排任务。

　　为确保实现上述目标，我们将重点抓好以下工作。

　　一、强化重建攻坚，科学推进灾后恢复重建

　　紧紧扭住重建中心不动摇，严格遵循重建总体规划和专项规划，全力加快重建进度，科学提升重建水平。

　　全面完成城乡住房重建任务。继续把城乡住房重建放在首位，加快做好重建农房配套设施建设，实现4月20日前全面乔迁新居;全力加快城房重建进度，12月31日前全面完成城房重建任务。

　　全力加快公共服务设施重建。把公共服务设施重建摆在优先位置，确保教育、医疗卫生类项目重建进度快于全市重建平均进度。秋季开学前全面建成基础教育(含幼教)重建项目并投入使用，板房学校学生实现全部搬离。12月31日前医疗卫生新建项目完工65%、改扩建项目完工100%，总体完工90%。加快文化项目重建，12月31日前完工80%。

　　切实抓好基础设施和城镇体系重建。以加快雅康高速公路和国道351线、318线、108线等道路交通项目建设为重点，统筹推进水利、能源等重大基础设施重建，不断改善发展条件。7月20日前完成国道108线改造;12月31日前基本完成国道351线多功至芦山县城段和国道318线主体工程建设，全面完成雅安城区至望鱼段、天全至芦山段经济干线建设。12月31日前，水利、能源重建项目分别完工70%和90%以上。按照新型城镇化要求，科学推进主城区、6个县城和16个重点镇重建项目建设，突出抓好“一城四镇”重建，12月31日前城镇体系框架基本形成。

　　扎实做好地质灾害防治。加快地质灾害治理工程建设。7月20日前，除重大地质灾害治理工程管理维护项目外，其余项目基本完成。坚持动态防治，加强监测预警，发挥“三大工程”、“三本台账”作用，避免因灾伤亡，最大限度减少损失。坚持自然修复与人工治理相结合，12月31日前完工生态修复项目90%以上。同步推进农村建设和土地利用项目重建，12月31日前分别完工85%和65%以上。

　　高水平推进产业重建。着眼雅安长远发展和群众致富奔康，把产业重建作为灾后恢复重建的重要内容，12月31日前产业重建项目完工80%以上。以工业园区建设为载体抓好工业重建，提升园区承载能力，加快企业退城入园，推动工业集约集聚发展。以发展现代农业为抓手加快农业重建，强化产业基地和园区建设，大力推进产业化经营。以生态文化旅游融合发展为重点推进服务业重建，一手抓精品旅游景点打造，一手抓乡村休闲旅游发展，催生一批旅游特色乡镇和旅游精品村。主动抓好对口援建项目实施，推动对口援建向全面合作转变;积极做好7个省级部门帮助招商项目对接，7月20日前开工项目达到30个以上。

　　科学提升重建整体水平。坚持和深化“五总”推进机制，规范重点区域、重要节点和重大项目建设管理。坚守“六位一体”工作底线，加强对项目安全、质量、资金使用等环节的全程监管，以“零容忍”的态度查处重建中出现的违法违纪行为，确保科学重建、阳光重建、廉洁重建。坚持“点、线、片”结合，整体提升重建水平，全面展示重建成果。把农房聚居点打造提升与幸福美丽新村建设和特色旅游发展结合起来，提升新村建设水平。把城房建设与新区建设、旧城和棚户区改造结合起来，着力打造一批独具特色的县城和重点镇。强化城乡环境综合治理，做好交通沿线重建项目风貌管控，推动重建项目周边环境整体提升。

　　强化重建体制机制创新。继续探索实践以地方为主的灾后恢复重建机制，系统总结完善灾后恢复重建的组织方式和推进模式，为国家出台指导性政策法规和省上系统研究做好基础工作。切实做好重建项目的竣工验收工作，建立健全重建成果管护运营机制，促进重建成果转化运用。

　　二、突出投资拉动，保持经济平稳较快发展

　　坚持经济工作项目化、项目工作责任化，进一步扩大投资规模，优化投资结构，强化项目管理，拉动经济平稳较快发展。

　　全力推进重点项目建设。实施市以上重大项目470个，完成投资315亿元左右。加快川藏铁路成都(朝阳湖)至雅安段建设，力争启动雨城客运站和名山货运站建设。加快铜头引水、九龙水库、南郊水厂引水工程建设，全面建成永定桥水利工程，实现向汉源县城供水。做好老鹰岩电站开工准备，开工建设锅浪跷电站主体工程，加快民治和出居沟电站建设进度，确保大岗山电站投产发电。

　　着力提高产业投资比重。在稳定投资增长的前提下，更加注重优化投资结构，着力引进并启动实施一批产业性投资项目，提高产业投资比重，增强发展后劲。把扩大工业投资作为优化投资结构的重中之重，完成工业投资165亿元以上、增长5%以上。积极引导房地产市场投资和消费，有步骤地推进存量土地投入开发，促进房地产行业健康发展。落实国务院“五个一律”要求，进一步激发民间投资活力。

　　不断提升项目工作水平。深入研究把握国家和省上政策动向，科学谋划一批事关全局、带动力强的重大项目。提高项目编制水平，高标准、高质量抓好项目库建设，形成“科学谋划一批、包装储备一批、争取引进一批、开工建设一批、竣工投产一批”的滚动发展局面，避免重建完成后项目投资出现回落。完善市级领导协调推进重大项目制度，强化协调推进责任，及时解决项目推进中存在的问题。积极搞好协调服务，强化要素保障，确保重点项目建设的用地、资金等需求。

　　三、坚持工业强市，推进工业经济增量提质

　　坚持调结构、转方式，进一步做大总量、提升质量，突出抓好重点园区、重点产业、重点企业，推动工业经济加快发展。

　　强化工业园区建设。着力提升园区承载能力，加快园区骨干路网、标准厂房等基础设施建设，完善服务配套。优化园区功能布局，引导企业向相适应的园区集约集聚发展。加强管理体制机制创新，探索园区管理服务“一体化”新模式。推进芦天宝飞地产业园区(雅安经开区)创建国家级经济技术开发区工作，加快芦山产业集中区、荥经循环经济示范园区建设发展，积极支持汉源、石棉省级工业园区和其他县级工业集中区、成雅工业园区健康发展。力争全市工业园区产值突破400亿元。

　　做大做强优势工业。持续推进“千亿产业”培育计划，以强化传统产业的可持续增长能力入手，着力壮大水电及矿电结合、机械装备制造、农产品加工等传统产业，形成稳定增长的产业支撑点;主动承接产业转移，发展壮大汽车生产制造、新能源新材料、生物制药、信息安全等成长型产业，培育新的经济增长点。力争水电及矿电结合产业产值达到240亿元以上，机械装备制造业、新材料新能源产业、农产品加工业产值均达到70亿元以上。全力加快恒天、建安、新筑通工、川西、三九、雅化、一名微晶、王老吉等重大产业项目建设。

　　支持企业转型升级。统筹用好灾后恢复重建产业发展基金、工业发展资金和留存电量等政策，重点支持重大产业项目建设、企业技术改造创新、企业科技研发和中小企业融资担保。引导企业加大技改投入，优化产品结构，提升产品质量，提高抗风险能力和核心竞争力，完成技改投资60亿元以上。支持企业加快建设国家和省级技术中心、工程中心、重点实验室等科研平台，构建以企业为主体、市场为导向、产学研相结合的技术创新体系。鼓励关联产业和上下游企业联合重组，支持优势骨干企业做大做强。扶持中小企业发展，稳步推进“助保贷”试点工作，着力解决好中小企业“融资难、融资贵”问题。新增规模以上工业企业20户以上，实现规模以上工业增加值增长9.5%;淘汰10户企业落后产能，万元工业增加值能耗下降实现省定目标。

　　四、转变农业发展方式，促进农村发展农民增收

　　以现代农业为方向，以改革创新为动力，切实加快农业现代化进程，促进农业持续增效、农民稳定增收、农村繁荣发展。

　　加快发展现代农业。按照“一三互动、农旅融合”的思路，发展壮大“5+3”优势特色产业，突出抓好百里茶产业、百里猕猴桃产业和百里花果金果产业带建设，新发展优势特色产业基地14.5万亩。强化农产品质量安全管理。积极推进绿色(有机)农业示范区建设，新发展绿色(有机)食品生产基地6万亩。抓好特色农产品精深加工和产业化发展，推动雅安国家农业科技园区扩区强园，完成国家验收评估工作，加强芦山现代生态农业示范园等园区建设，实现产业化龙头企业销售收入增长10%以上。重点加快雅茶和雅竹产业发展，推动蒙顶山茶和藏茶产业做大做强，不断扩大品牌知名度;努力在引进林竹精深加工龙头企业上实现突破，促进林竹资源就地转化利用。

　　强化农村改革创新。稳定土地承包关系，依法保障农民对承包地占有、使用、收益、流转及承包经营权抵押、担保权利。深化集体林权制度改革。统筹推进农村各类产权“多权同确”，重点推进土地承包经营权确权登记颁证并完成工作量的60%以上，全面完成雨城、石棉、汉源3个县(区)农村房屋所有权确权登记颁证试点。积极推进土地经营权流转，着力培育一批专业大户、家庭农场、农民合作社、农业企业等新型农业经营主体，鼓励发展多种形式适度规模经营。加强与四川农业大学等高等院校、科研院所的合作，完善新型农村科技服务体系。积极探索农民财产权实现形式，有序推进农民财产性收入改革试点。强化农民技能培训，拓展农民增收渠道。

　　夯实农村发展基础。稳定粮食生产能力。积极支持现代农业、畜牧业、林业产业重点县建设。加强农田水利基本建设，提升农业综合生产能力，新建高标准农田4.5万亩，新增和恢复灌溉面积15.53万亩，解决18万人安全饮水问题，建设农村公路368公里、通组公路(林区道路)1000公里，增设农村公路路侧护栏160公里。新建幸福美丽新村100个，统筹抓好旧村落改造和传统村庄院落民居保护。

　　五、发挥特色优势，加快现代服务业发展

　　将提高服务业比重作为调整优化经济结构的重要着力点，重点加快现代服务业发展，促进服务业发展提速、水平提升。

　　突出发展生态文化旅游业。围绕建设国际生态文化旅游目的地、中国西部康养目的地、川西休闲度假旅游集散地，大力推进生态文化旅游融合发展试验区建设。加快碧峰峡、蒙顶山创建国家5a级旅游景区工作，大力支持芦山龙门古镇、宝兴硗碛藏寨、天全喇叭河、汉源九襄花海果乡创建国家4a级旅游景区，积极推进牛背山、汉源湖、孟获城、达瓦更扎等重点旅游项目开发，改造提升雅安城区至上里、荥经城区至龙苍沟、泗坪至牛背山等重要旅游通道。加强与周边地区的旅游协作和线路对接，主动融入“大峨眉”、“大香格里拉”、“大九寨”等区域旅游大环线。办好第十一届蒙顶山国际茶文化旅游节等活动，强化旅游市场管理，实现旅游综合收入增长16%。

　　大力发展现代商贸服务业。加快推进大型城市商业综合体建设，积极引进国内外高端品牌和知名商贸流通企业入驻雅安。培育发展姚桥新区集零售、餐饮、酒店、休闲为一体的现代购物休闲中心，增强城市服务功能。加强特色商业街的建设改造，重点打造西门茶廊、茶马古城、一品天下等体现地方文化特色的商业街区。加强各类专业市场建设，逐步打造川西商贸中心。强化农村商贸服务体系建设，改善农村消费环境。

　　积极发展新兴先导型服务业。发展现代物流业，着力构建“一园区、四中心、三节点”现代物流体系，培育发展第三方物流企业。发展现代金融业，推进金融产品服务创新，强化金融市场监管，规范小额贷款和融资性担保公司管理。发展健康养老服务业，转化利用雅安生态资源优势。发展电子商务业，引导企业、超市开展电子商务交易，力争在200个村建立电子商务服务站。

　　六、深入推进改革开放，增强发展的动力和活力

　　坚持激发内力与借助外力并举，通过深化改革强动力，通过扩大开放添活力，形成加快发展的强大合力。

　　深化经济体制改革。深化财税体制改革，改进预算管理，继续推进“营改增”扩围试点。深化金融领域改革，支持雅安农村商业银行深化改革，做好雅安市商业银行增资扩股工作;拓宽直接融资渠道，推动具备条件的企业做好上市工作。深化国有企业改革，全面推进市属国有企业重组整合，启动市属国有企业依法选聘管理层改革试点，合理确定和严格规范国企管理人员薪酬水平、职务待遇和消费标准。深入推进蒙顶山茶叶交易所股权改革。有序推进资源性产品价格改革，建立健全天然气阶梯价格制度。完成机关事业单位养老保险并轨。

　　提升开放合作水平。抢抓国家推动“一带一路”和长江经济带发展等政策机遇，积极融入国家对外开放大格局。主动融入“1+7”同城化发展，深化与周边市(州)的合作，积极参与攀西战略资源创新开发试验区建设。强化招商引资项目库建设，建立完善重大项目统筹推进机制和招商引资信息共享协调机制。精心组织参加“中外知名企业四川行”和“川欧合作”等投资促进活动，努力提高引资实效。坚持重落地、重投产、重带动，力争在汽车生产制造、新材料新能源、生态文化旅游、现代商贸物流、生物制药、农产品加工等领域引进一批竞争力强、成长性好和关联度高的项目。引进到位市外资金325亿元以上，引进投资5亿元以上项目10个;实现进出口总额7930万美元以上，实际利用外资2000万美元以上。

　　加快县域经济发展。着力打造“三带一核一圈五廊”〔注20〕，优化县域发展空间格局。坚持县域联动、特色发展，支持县(区)转化比较优势，强化县域产业支撑。全面落实扩权强县和支持县域经济发展的各项政策，在发展战略制定和项目、资金、政策等方面加大帮助、指导、服务力度，充分激发县域发展活力。积极支持石棉县推进独立工矿区转型发展试点工作。进一步创优政策，营造环境，放手发展民营经济，力争民营经济增加值占地区生产总值比重提高1个百分点左右。积极扶持小微企业发展，将符合条件的“个转企”企业纳入小微企业扶持范畴，加大信贷、税收及工商注册登记等支持力度。充分发挥行业协会、商会的作用，切实关爱民营企业家成长。

　　七、加快推进新型城镇化，着力构建城乡一体发展格局

　　按照雅安城市定位，科学完善城镇规划体系，同步加强城镇建设管理，不断提高城镇化水平。

　　科学完善城镇规划体系。完善姚桥新区、名山城区和芦天宝飞地产业园区(雅安经开区)等控制性详细规划及重要节点城市设计。完成石棉、汉源县城总体规划及蒙顶山风景区总体规划修编和报批工作。启动编制历史文化名城、镇(村)保护规划。科学编制综合交通规划、市政基础设施等专项规划。扎实推进重点乡镇规划编制，进一步完善城乡规划体系。

　　全力加快城镇建设步伐。坚持新区建设与旧城改造并举，着力提升城市形象，改善群众居住生活条件。突出抓好主城区“一城两线三出口”改造提升，全面完成成雅高速公路姚桥、北郊、多营进出口大道改造，完善姚桥新区城市功能和雅州大道、熊猫大道沿线的配套设施，实现北外环线全线通车。全力推进水中坝整体开发建设。全面加快大兴组团道路、管网等基础设施建设。科学规划建设火车站站前商务区、市体育文化中心区、青衣江大桥商务休闲区周边市政基础设施。大力推进雨名连接线等基础设施建设，促进雨城区、名山区和芦天宝飞地产业园区(雅安经开区)产城一体化发展。支持名山新区建设，新建蒙顶山大道，改造皇茶大道和茶都大道。支持县城和重点镇建设，进一步完善城镇设施，提升承载能力，吸引人口适度集聚。

　　切实提升城镇管理水平。加快创建省级文明城市，积极争创国家级卫生城市。坚决维护规划的权威性、统一性和完整性，依法整治违法建设行为。优化城市路网，治理易堵路段，提高通行能力。强化园林建设管护，提升城市绿化水平。加快智慧城市建设，建成数字化城市管理信息系统。完善城市管理考核机制，加强对背街小巷、城乡结合部、农贸市场等重点区域的治理，重点整治城市“五乱”现象。加强县城、场镇和农村环境治理，全面开展城乡生活垃圾分类收集和处理，持续改善群众生活环境。

　　八、大力保障和改善民生，保持社会和谐稳定

　　把保障和改善民生作为工作的出发点和落脚点，积极回应民生诉求，切实加大民生投入，用心办好民生实事，重点实施好“十项民生工程”和“20件民生实事”。

　　大力发展社会事业。优先发展教育事业。继续推进中小学标准化建设，加快农村薄弱学校特别是中高山区和民族地区学校建设，促进义务教育资源均衡配置;提升普通高中教育质量，加快职业教育发展区建设，扩大学前教育覆盖面，办好特殊教育和继续教育;深化教育综合改革，强化教师业务能力和师德师风建设，进一步解决优秀教师和优质生源流失问题;加强校园安全管理。大力发展卫生计生事业。推进服务重心下移，进一步健全市、县、乡、村四级医疗卫生计生服务体系;深化医药卫生体制改革，重点实施县级公立医院综合改革;全面实施分级诊疗制度，让有限的医疗资源得到合理利用;坚持计划生育基本国策，稳步实施“单独两孩”政策。加快发展文化体育和广电新闻出版事业。强化文化基础设施建设，繁荣文艺创作，丰富群众文化生活;加强优秀传统文化传承和文化遗产保护利用，做好第一次全国可移动文物普查工作;积极组团参加首届四川艺术节、第五届中国成都国际非遗节;大力开展全民健身活动;完成市广播电视台和雨城区电视台资源整合。深化科技体制改革，促进产学研协同发展。做好全国第二次地名普查工作。全面发展社会福利、慈善、妇女儿童、残疾人、红十字和老龄事业。重视做好防震减灾、地方志、档案、保密、社会科学、外事、侨务、对台等工作。

　　着力健全社会保障。抓好就业促进工作，城镇新增就业1.6万人。完善各类困难群体救助机制，稳步提高城乡低保和“五保”供养水平。实行80周岁以上高龄老人生活津贴制度。城乡居民养老保险、城镇职工养老保险分别覆盖39万人和33万人。巩固扩大城乡医保覆盖面，启动实施医疗责任保险，推进大病保险和异地就医及时结算。开工建设保障性住房和实施危旧房棚户区改造3518套，竣工3273套。积极争取国家和省政策扶持，加快贫困地区、革命老区、少数民族地区发展。完善精准扶贫帮扶机制，加大对贫困村、贫困户的帮扶力度。立足库区跨越发展和长治久安，持续抓好库区后续建设、管理、发展、稳定工作。

　　深入推进依法治理。深入开展“六五”普法，提高全民法治意识。大力推进法治雅安、平安雅安建设，加强社会治安综合治理和网格化服务管理，强化重点区域、重要目标安全保卫，严厉打击违法犯罪。用法治思维和方式化解社会矛盾，进一步整合人民调解、行政调解、司法调解力量，推动“大调解”工作向纵深发展。加强基层政权建设，创新社会治理体制和群众工作方法，支持群团部门参与社会治理，及时化解社会矛盾，实现政府治理和社会自我调节、居民自治良性互动。强化安全生产依法治理，加强煤矿、非煤矿山、建筑、消防、道路交通、危化、特种设备等重点领域安全监管，坚决遏制重特大安全事故。继续推进煤矿整顿关闭和兼并重组工作。建立完善覆盖全过程的食品药品安全监管制度，构建专业、高效的食品药品安全监管体系。完善应急管理体系，提升应急处置能力。加强国家安全、民族、宗教、人民防空、国防动员和民兵预备役等工作。

　　九、推进生态文明示范建设，增强可持续发展能力

　　将生态文明理念融入经济社会发展全过程，扎实推进国家首批生态文明先行示范区建设，促进人与自然和谐发展。

　　强化生态环境保护。实行最严格的水资源管理制度，抓好青衣江、大渡河等流域水污染治理和饮用水源保护工作，重点抓好主城区“一江三河”〔注21〕水污染治理。继续做好空气质量常规监测，完成雅安市环境空气质量信息集成平台升级建设，定期面向社会发布空气质量。强化工业企业、城市扬尘污染监测治理。整治农村面源污染，推进行政村环境连片整治，加强农村生活垃圾和污水处理。开展环境保护大检查，落实行政区域划分环境监管网格化制度，重拳打击环境违法行为。

　　强化生态工程建设。深入推进“三大生态工程”，森林覆盖率达到63%。坚守耕地保护红线，提高国土资源节约集约利用水平，做好土地开发整理。加强自然保护区、森林公园、风景名胜区和湿地资源保护，推进大、小相岭大熊猫生态放归基地建设。强化水土保持，治理水土流失面积105平方公里。加强石漠化治理。开工建设城市生活垃圾处理厂和大兴污水处理厂。

　　强化生态资源转化。积极发展绿色经济、生态经济、循环经济，加快把生态资源优势转化为生态经济优势。严格环境准入，严控高耗能、高污染和产能过剩行业。加大技术改革创新，加强资源循环利用，促进循环经济形成规模和特色。

　　在抓好今年各项工作的同时，科学做好“十三五”规划编制，引领“十三五”全市经济社会平稳健康发展。

全面加强政府自身建设

　　各位代表，我们深深地知道，新形势、新任务对政府工作提出了更高的标准和要求。我们将主动适应形势发展需要，顺应人民群众期盼，全面加强政府自身建设，奋力开创政府工作新局面。

　　我们将更加注重依法行政，全面加强法治型政府建设。认真贯彻落实党的十八届四中全会、省委十届五次全会和市委三届八次全会精神，维护宪法和法律的权威，严格按照法定权限和程序行使权力、履行职责。自觉接受市人大及其常委会的法律监督、工作监督和市政协的民主监督，认真执行市人大及其常委会的决议决定，认真办理人大代表建议和政协委员提案。认真听取民主党派、工商联、无党派人士和人民团体的意见建议，开放接受人民群众和新闻舆论监督。健全依法决策机制，坚持重大决策专家论证、公众参与、风险评估、合法性审查等制度。强化行政执法规范化建设，落实行政裁量基准制度，清理规范执法项目。建立健全行政执法责任制，提升执法人员法律素质和能力。

　　我们将更加注重简政放权，全面加强服务型政府建设。发挥市场在资源配置中的决定性作用，科学处理好政府与市场、政府与社会的关系，加快转变政府职能。深化行政审批制度改革，对行政审批事项实行目录管理，加快建设权力清单、责任清单和负面清单，推进以“事前审批为主”向以“事中事后监管为主”转变。加快整合政务服务平台与公共资源交易平台，强化“两集中、两到位”〔注22〕，大力推进并联审批、网上审批，切实提高行政服务效率和水平。依法规范和深化政务公开，推进财政预算、公共资源配置、重大建设项目、公益事业建设等政府信息公开。

　　我们将更加注重从严治政，全面加强廉洁型政府建设。坚持把从严治党与强化政府自身建设有机结合，以纯政风、严政纪为重点，全面加强公务员队伍建设。着力巩固党的群众路线教育实践活动成果，进一步健全密切联系群众和改进作风的常态化机制，持续治理“庸懒散浮拖”等现象。完成全市党政机关公车制度改革。严格落实党风廉政建设责任制，健全廉政风险防控体系，加强行政监察和审计监督，强化对重点领域和关键岗位权力运行的监督。旗帜鲜明地反对腐败，加大违法违纪案件查处力度，以反腐倡廉的实际成效取信于民。

　　各位代表，让我们更加紧密地团结在以习近平同志为总书记的党中央周围，在省委、省政府和市委的坚强领导下，始终保持昂扬向上的斗志、攻坚克难的决心、雷厉风行的作风，团结带领全市人民，聚精会神抓重建，一心一意谋发展，为奋力促进雅安经济社会发展新跨越，加快建设幸福美丽新家园而努力奋斗!
